# Fault-controlled deep hydrothermal flow in a back-arc tectonic setting, SE Tyrrhenian Sea

**DOI:** 10.1038/s41598-019-53696-z

**Published:** 2019-11-27

**Authors:** Maria Filomena Loreto, Doğa Düşünür-Doğan, Serkan Üner, Yeliz İşcan-Alp, Neslihan Ocakoğlu, Luca Cocchi, Filippo Muccini, Patrizia Giordano, Marco Ligi

**Affiliations:** 1 Istituto di Scienze Marine, CNR, Via P. Gobetti 101, 40129 Bologna, Italy; 2 Department of Geophysical Engineering, Faculty of Mines, Istanbul Technical University, Maslak, 34469 Istanbul Turkey; 3 Department of Geophysics, Faculty of Engineering, Istanbul University, Avcılar, 34850 Istanbul Turkey; 4 Istituto Nazionale di Geofisica e Vulcanologia, Via di Vigna Murata 605, 00143 Roma, Italy; 5 Istituto di Scienze Polari, CNR, Via P. Gobetti, 101, 40129 Bologna, Italy

**Keywords:** Geodynamics, Geophysics

## Abstract

Understanding magmatic systems and deep hydrothermal circulation beneath arc-volcanoes provides insights into deep processes associated with slab-subduction and mantle-wedge partial melting. Here we analyze hydrothermal flow below a structural high (Capo Vaticano Ridge, CVR) located offshore Capo Vaticano (western Calabria) and affected by magmatic intrusions generated from above the Ionian subducting-slab. In order to explain observations, we combine geophysical and numerical modelling results. Fluid-flow modelling shows that temperature distribution and geothermal gradient are controlled mainly by hydrothermal circulation, in turn affected by heat source, fault pattern, rock permeability, basement topography and sediment thickness. Two main faults, shaping the structural high and fracturing intensely the continental crust, enable deep hydrothermal circulation and shallow fluid discharge. Distribution of seismicity at depth supports the hypothesis of a slab below Capo Vaticano, deep enough to enable mantle-wedge partial melting above the subduction zone. Melt migration at shallow levels forms the magmatic intrusions inferred by magnetic anomalies and by δ^3^He enrichment in the discharged fluids at the CVR summit. Our results add new insights on the southern Tyrrhenian Sea arc-related magmatism and on the Calabrian inner-arc tectonic setting dissected by seismogenic faults able to trigger high-destructive earthquakes.

## Introduction

Although large steps forward have been achieved on our understanding of hydrothermal processes, deep fluid circulation is still not fully explored. Likewise, a complete view of volcanic plumbing systems is hard to obtain, mainly due to their complexity and to the difficulties in obtaining direct information. Some authors suggested that magma upwelling is commonly controlled by tectonics, namely normal faults, strike-slip faults or, even if rarely, inverse faults^1,2^. Normal faults are more frequently associated to volcanism and hydrothermal fluids^3^ due to the prevailing extension controlling the release of lithostatic pressure within rocks^4–6^. Furthermore, geochemical interaction can be activated among cooling magmatic rocks, hosting rocks and hydrothermal fluids^7–11^.

Subaerial volcanoes, back-arc systems and mid-ocean ridges have been widely investigated in terms of hydrothermal vents distribution and circulation pattern by bathymetric and backscatter, magnetics, electro-magnetics, gravity and seismic methods^12–15^. High-resolution multibeam bathymetry constrains spatially hydrothermal activity^6,16^, while vent chemistry helps to determine the source of hydrothermal fluids as well as fluid crustal residence time related to rock permeability^17^. However, the lack of direct information, such as drilling, makes numerical modelling an important tool in understanding fluid/rock interactions and hydrothermal circulation within an active tectonic setting^18,19^. Modelling of deep fluid circulation constrained by geophysical data has already been proposed, as for instance that based on Self Potential (SP) and/or electromagnetic data proposed for subaerial volcanoes^20,21^, also part of arc/backarc systems^22–24^.

**Figure 1:**
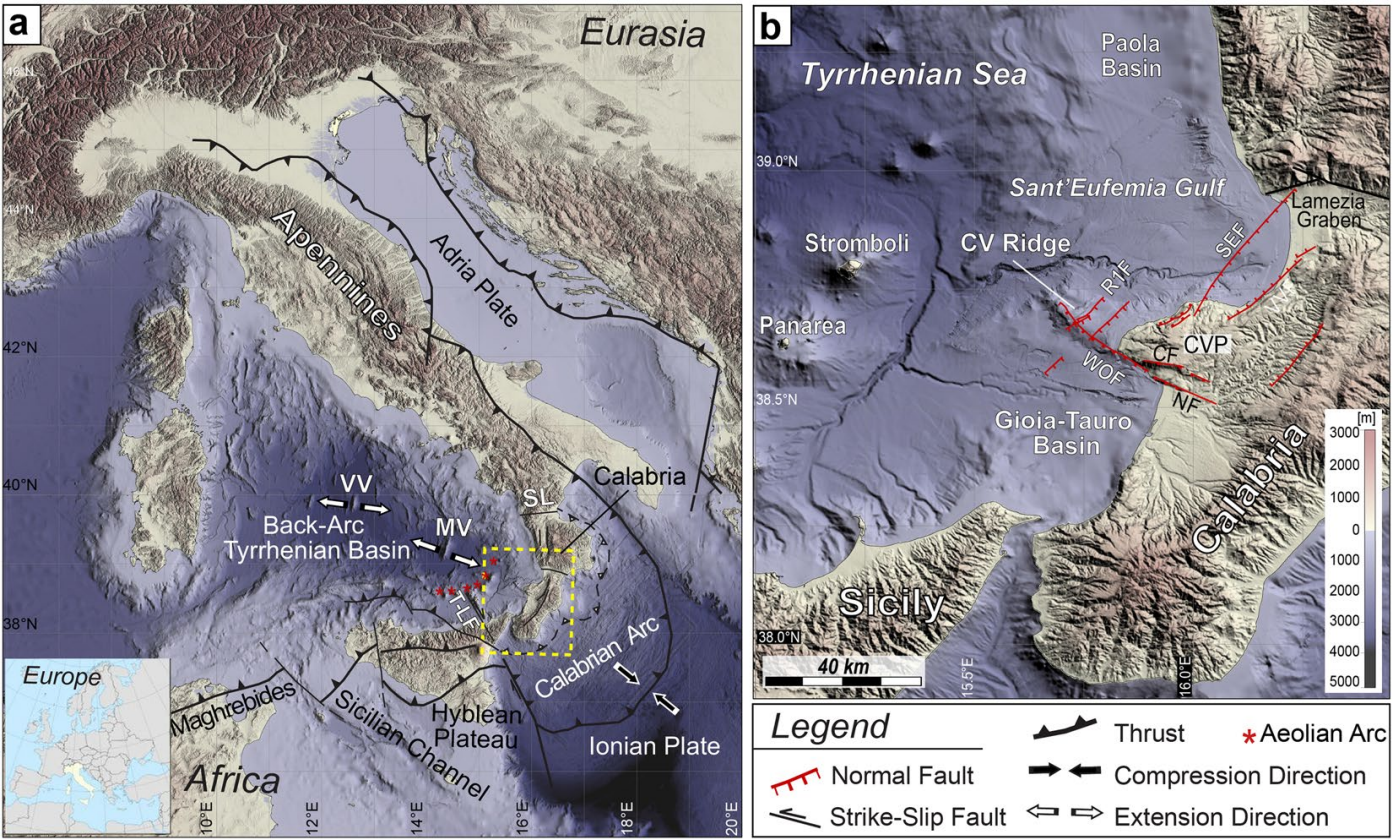
Structural setting of the study area. (**a**) Topographic map of Italy including a tectonic sketch of the Apennine Belt (structural lineaments based on refs. ^34,41^) obtained with the GMT package^80^ using the SRTM30 Plus (*David T*. *Sandwell*, *Walter H*. *F*. *Smith*, *and Joseph J*. *Becker*. *The Regents of the University of California*. *All Rights Reserved*) and the EMODnet bathymetry (*EMODnet Bathymetry Consortium*, *2016: EMODnet Digital Bathymetry*, http://doi.org/10.12770/c7b53704-999d-4721-b1a3-04ec60c87238) datasets. The yellow dashed box outlines the location of morphological details shown in (**b**). Red asterisks indicate locations of subaerial volcanic edifices of Aeolian Arc. VV: Vavilov Volcano; MV: Marsili Volcano; SL: Sangineto Line; T-LF: Tindari-Letojanni Fault. (**b**) Simplified structural map of the Central-Western Calabria and its offshore. Swath bathymetry data from ISTEGE project (2010) combined with 250 m EMODnet and 90 m SRTM grids.

**Figure 2:**
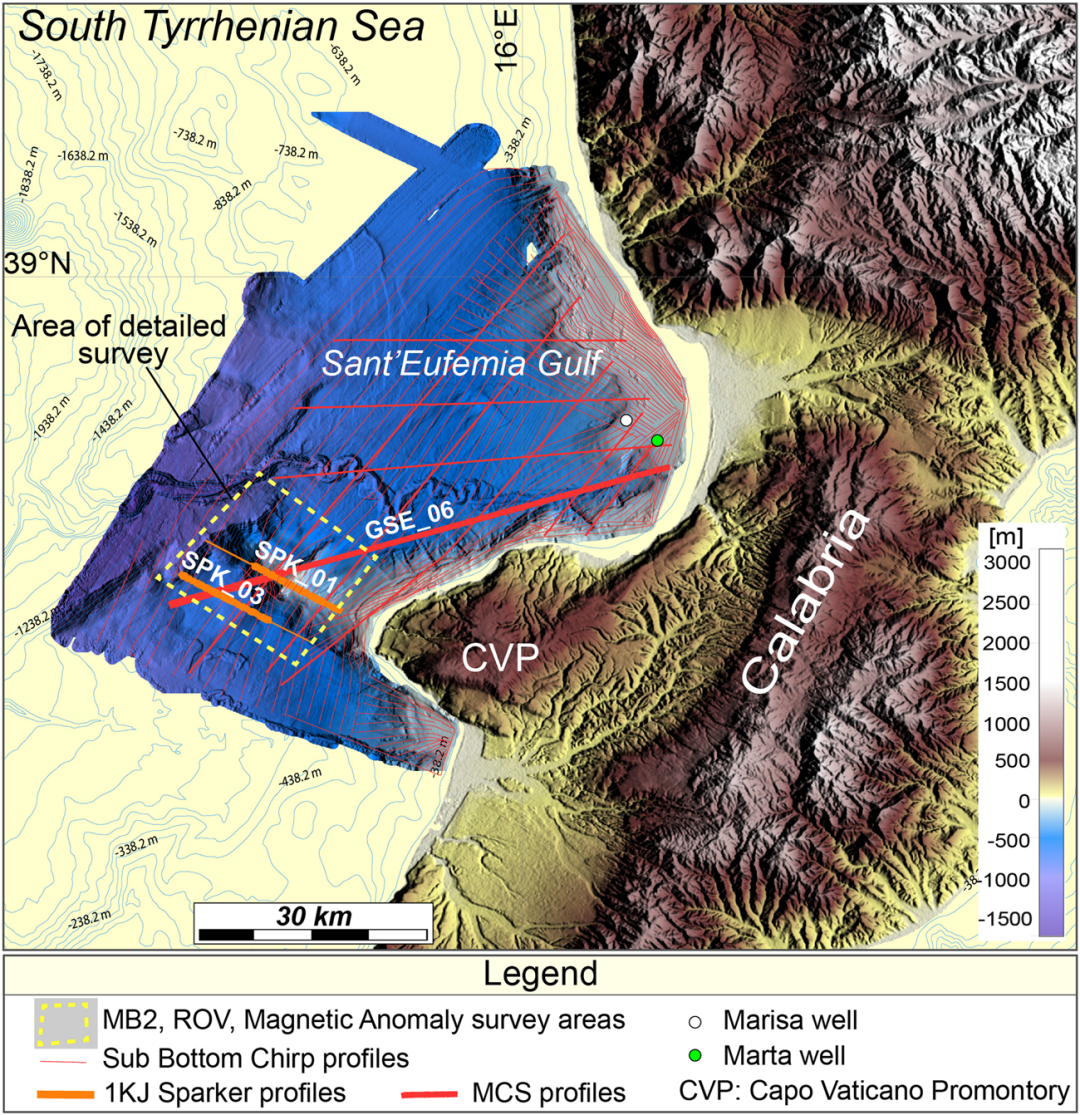
Morphology of the study area. Shaded relief image obtained with the GMT package^80^ from swath bathymetry dataset MB1 (ISTEGE 2010) combined with the SRTM30 onshore dataset (*David T*. *Sandwell*, *Walter H*. *F*. *Smith*, *and Joseph J*. *Becker*. *The Regents of the University of California*. *All Rights Reserved*). The map is complemented with contours (cyan thin lines) from the EMODnet bathymetry dataset (*EMODnet Bathymetry Consortium*, *2016: EMODnet Digital Bathymetry*, http://doi.org/10.12770/c7b53704-999d-4721-b1a3-04ec60c87238). Thin red lines indicate the location of swath bathymetry profiles acquired during the ISTEGE 2010 cruise. Thick red line shows the location of multichannel seismic profile GSE_06 shown in Fig. 3. Thick orange lines indicate the location of single channel sparker profiles SPK_01 and SPK_03 shown in Fig. 4. The yellow dashed box outlines the location of high resolution shipborne magnetic survey shown in Fig. 5. MB2: MultiBeam dataset acquired during the Geocal 2014 survey.

This work aims to analyze a submarine structural high located offshore Capo Vaticano along the western coast of Calabria (SE Tyrrhenian Sea), named Capo Vaticano Ridge (Figs. 1 and 2), where shallow magma injections, probably fed by a deep seated source, and hydrothermal circulation has been suggested. The tectonic setting of this structure is complicated by the presence of two main faults: the Western Offshore Fault^25^ (WOF), an extensional fault that controlled the SW-deepening of the adjacent Gioia-Tauro basin from Pliocene to Pleistocene; and the Ridge 1 Fault^26^ (R1F), a NE-SW oriented normal fault that crosses the eastern flank of the CVR. Based on aeromagnetic data, this structural high has been interpreted as a remnant of a Pleistocenic volcanic edifice^27^. Indeed, the presence of magmatism in the area is suggested by evidence of hydrothermal vents and by the geochemical anomalies of seawater sampled at the summit of the CVR^28^. However, magmatic rocks have never been recovered from the CVR and the only volcanic products believed to be generated by this hypothetical volcanic apparatus are pumices trapped within the onshore sediments of the Gioia-Tauro basin^27,29^. In addition, volcanic features such as craters, small cones or lava flow morphologies have never been observed along the flanks of the CVR by swath bathymetry data^25,26^. An alternative scenario could be that the observed seawater geochemical anomalies at the summit of the CVR are due to cold seep related fluid flow. However, the lack of a thick sedimentary cover over the CVR summit (less than 200 m)^26^, the lack of morphological evidence for flares and pockmarks^25,26^, the strong magnetic anomaly^27^ and the high content of mantle derived ^3^He in the vent escaping fluids^28^ prevent to follow this hypothesis.

Forward and inverse magnetic modeling combined with seismic and morpho-bathymetric images defines some features of the magnetized body at depth and outlines the ongoing tectonics shaping the ridge^26^. If on the one hand previous studies suggest the presence of magmatic rocks just below the top of the tectonically-controlled CVR^26^, on the other hand they open some questions: for instance, is the CVR the remnant of a volcanic complex that erupted during Pleistocene time? Alternatively, are the observed magnetic anomalies related to shallow dyke intrusions fed by a deeper magmatic source, with magma never having reached the surface? What is the structure of the magmatic plumbing system and where is it rooted? In order to answer these questions, we carried out numerical modeling of hydrothermal fluids and interpreted results together with all available geophysical data.

## Geological Background

The Capo Vaticano Ridge is located at the transition between the Tyrrhenian back-arc basin and the Apennine chain (Fig. 1a). This is one of the most active areas of the entire Apennine system, due to the Ionian lithosphere subduction under the Calabrian Arc and to the inner arc collapse accommodated by several normal faults: a structural complexity suggested by the depth distribution (from 0 to 400 km) of the instrumental seismicity recorded in the area. The Calabrian arc is part of the Apennine-Maghrebide fold-and-thrust belt, formed by a stack of metamorphic and sedimentary units that include Hercynian and pre-Hercynian continental basement and Jurassic to Early Cretaceous Ophiolite sequences^30,31^. This region, originally part of the Corsica-Sardinia block, separated from it during a first Tortonian extensional phase, and drifted southward carrying the nascent Apennine belt on its top^30,32–34^. The following E and SE-ward roll-back and retreat of the Ionian slab drove the opening of the Tyrrhenian back-arc basin^32^ with the formation of the Magnaghi, Vavilov and Marsili volcanic complexes^35^, and the migration of the associated volcanic arc toward SE.

The present day subducting lithosphere defines a 70°-dipping Wadati-Benioff zone^36^, that under a given range of temperature and pressure conditions favoured by dehydration of the sediments above the slab, triggers mantle wedge partial melting^37,38^, resulting in the formation of the Aeolian Volcanic Arc^39,40^. The several volcanoes of the Aeolian arc are arranged in a ring shape around the Marsili volcano, west and north of Calabria and Sicily, respectively (Fig. 1a). Among the active volcanoes, the Stromboli volcanic complex lies only 70 km away from the Capo Vaticano Promontory (Fig. 1b). Finally, the modern subducting slab is bounded by two main tear faults (Subduction Transform-Edge Propagators, STEP) (Fig. 1a): (1) the Tindari-Letojanni fault to the south^41^; and (2) a main northern fault not uniquely defined, i.e., the Sangineto Line^41^ with its offshore prolongation beneath the Palinuro volcanic complex^42^; or the more southern Catanzaro shear zone^43^.

Extensional and strike-slip faults fragment longitudinally (from N-S to SW-NE) and transversally (from E-W to ESE-WNW) the Calabrian block^44,45^. The N-S to SW-NE-trending normal faults accommodate the rapid uplift of the Calabrian belt since middle Pleistocene time^43,45,46^, in particular along the western side of the belt (western Calabria), where widespread subsidence is observed^47^. Normal faults extend also offshore forming partially submerged graben or half-graben systems, such as the Lamezia graben^43,48^. The ESE-WNW trending transcurrent faults, including the WOF, accommodate differential movements of minor blocks^32^ since middle Miocene^49^. The presence of deep ESE-WNW oriented Pleistocene basins^50^ suggests strike slip systems with strong extensional component.

## Results

### Structural constraints from seismic profiles

**Figure 3:**
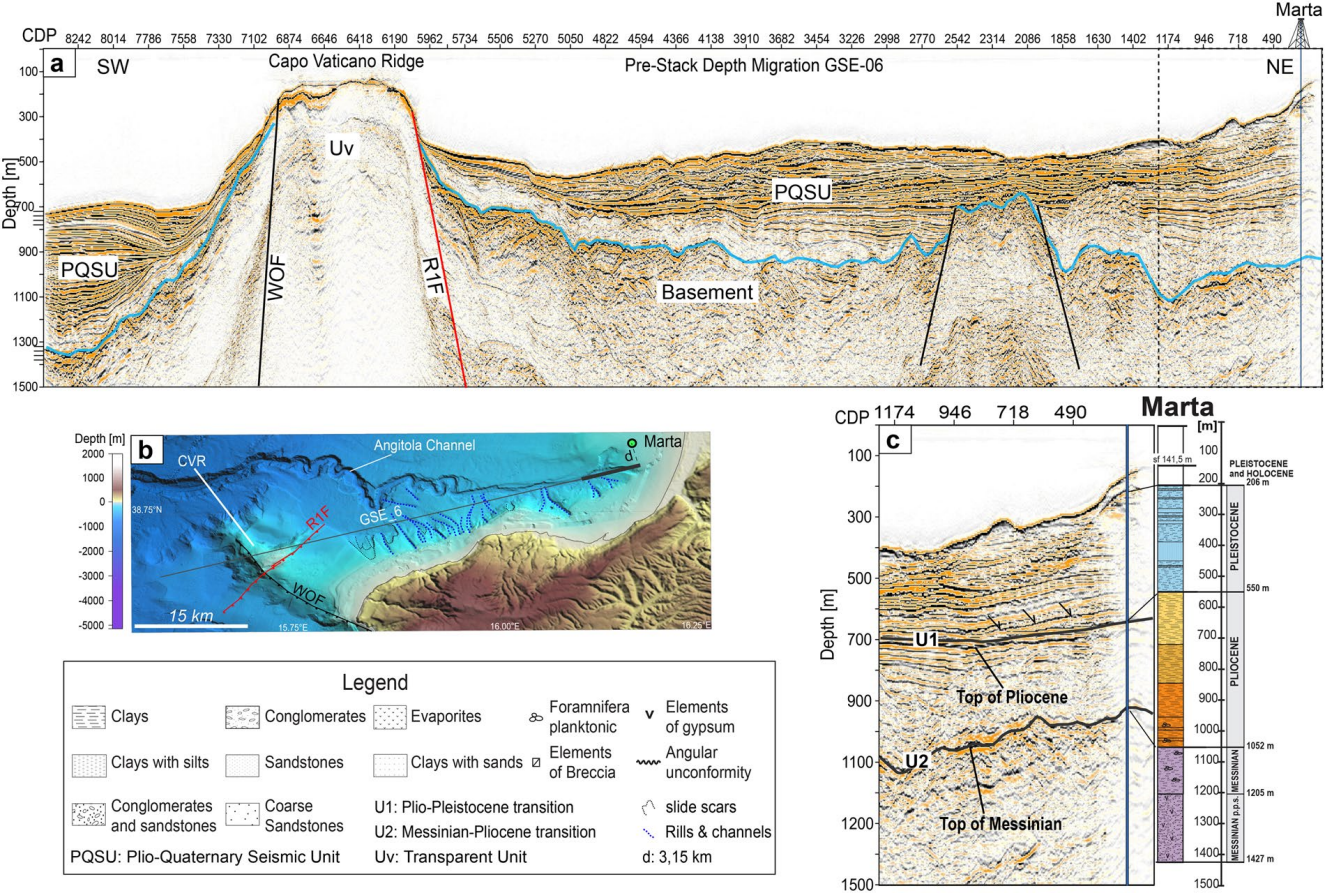
Geological section crossing Capo Vaticano Ridge. (**a**) Pre-stack depth migrated image of seismic line GSE_06 crossing S. Eufemia Gulf and Capo Vaticano Ridge. Thick cyan line marks the base of the Plio-Quaternary sedimentary sequence. (**b**) Morpho-bathymetric map obtained with GMT package^80^, showing the main morphological features of the area, the GSE_06 seismic profile and the Marta borehole locations. (**c**) Marta oil-well (thick black line in **b**) stratigraphy projected into a portion of the GSE_06 (black dashed line in **b**). U1 and U2: unconformities; sf: seafloor.

Faults geometry and sediment thickness were obtained from depth migrated seismic profile GSE_06 (Fig. 3a, see Methods for acquisition and processing details). We calibrated the seismo-stratigraphic units using the Marta well, drilled in the inner part of S. Eufemia Gulf (Fig. 2) by the Eni S.p.A. oil company. The small misfits between the borehole stratigraphy and the main seismic reflectors are due probably to the seismic line not crossing the borehole, located northward at a distance of 3.15 km (Fig. 3b). We identified three main seismic units from Messinian to present. The top of Messinian corresponds to a high amplitude unconformity named U2 (~950 m deep at the well site) that bounds below with a poorly reflective and chaotic unit; and above with a more stratified, although less reflective, Pliocene sedimentary unit (Fig. 3c). This last is delimited at its top by another angular unconformity named U1 (~600 m deep at well location). Above U1, well-stratified horizons terminate down-dip (Fig. 3c) with downlap truncations^51^. This unconformity corresponds to the Pliocene-Pleistocene, transition whose seismic character is well recognized in the sedimentary basins offshore of western Calabria^25,26,48,52^ and onshore of Catanzaro paleo-strait^50^. The detailed lithology of these units is shown in the legend of Fig. 3. Within the highly reflective and stratified Pleistocenic sequence, reflectors are interbedded with chaotic material. This is due to the location of the MCS profile that runs sub-parallel to the Angitola channel crossing slope deposits affected by channels and slides/slumps (Fig. 3b); features that move sediments from the coastal area to the basin. Along the entire section, the Plio-Quaternary sequence (PQSU) shows large lateral thickness variations, particularly SW of the CVR where it is ~650 m thick, while to the NE it is only ~200 m thick (Fig. 3a).

**Figure 4:**
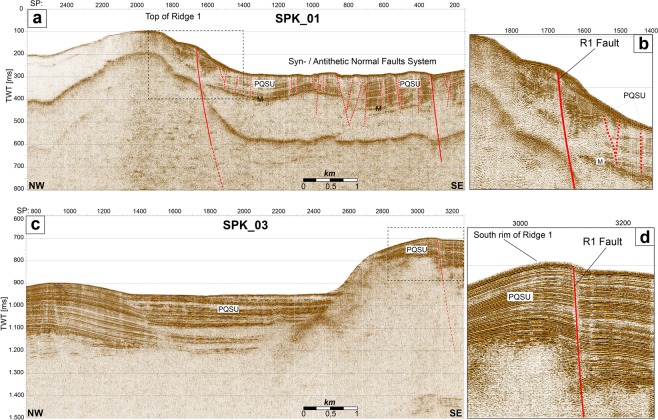
Fault pattern. (**a**) SPK_01 1 KJ sparker profile, see Fig. 2 for location. Dashed box outlines the detail shown in (**b)**. (**b**) Details of R1F offsetting the entire sedimentary sequence culminating with wedge shape deposits interpreted as part of a low-stand system tract formed during the Last Glacial Maximum. (**c**) SPK_03 seismic profile. Dashed box outlines the detail shown in (**d)**. (**d**) Details of R1F offsetting the Plio-Quaternary Sedimentary Unit.

Along profile GSE_06 we interpreted two structural highs both bounded by a couple of main extensional faults (Fig. 3a). The north-easternmost high is buried below 300 m-thick Pleistocene sediments and is located within the S. Eufemia Basin that corresponds to the southern tip of the Paola basin (Fig. 2). The continuity of reflectors above the structural high suggests that the two bounding faults ceased their activity since the end of Pliocene. Although the depth of these faults is not well constrained by the seismic profile, we suggest that the faults do not cut the entire pre-Messinian sequence reaching the maximum depth of ~1.3 km. Offshore Capo Vaticano, the PQSU and the Messinian sequences on-lap both flanks of the southernmost structural high (CVR) imaged by a transparent unit (Uv) with a specific seismic response. The two bounding faults cut the entire sedimentary sequence up to the seafloor. These faults are the WOF, presently not active^25^, and the R1F, probably active^26^. Sparker profiles (Fig. 4) acquired across the structural high (Fig. 2) delineate the trend of the R1F. This sub-vertical normal fault is revealed by the interruption and rotation of well stratified sediments on the eastern part of the Ridge 1 (Fig. 4). This sedimentary sequence has a wedge shape (Fig. 4a,b). Given the present depth of these sediments, we interpreted them as part of a low-stand system tract deposited on the continental shelf at the end of Pleistocene during the Last Glacial Maximum^25,53^. The R1 fault plane reaches the seafloor as evidenced by the sharp scar in the gridded swath bathymetry (Fig. 2). Southeastward, the Plio-Quaternary sediments are affected by a set of minor faults organized in a synthetic and antithetic system, confined by a SE-dipping major fault (SP 340). To the South, R1F offsets the seafloor, the entire PQSU and also the underlying units (SPK_03; Fig. 4c,d). Accordingly, R1F is a NE-SW oriented Holocene normal fault, part of the extensional system currently deforming the Calabrian Arc.

Combining bibliographic information^25,54^ with the high resolution morpho-bathymetry data, we determined the geometry of these two faults. WOF strikes SE-NW, it is 50 km long and according to ref. ^55^ has an estimated down-dip width of 22 km, while R1F strikes SW-NE and is only 15 km long with an estimated depth of 12 km.

### Magnetic data analysis

**Figure 5:**
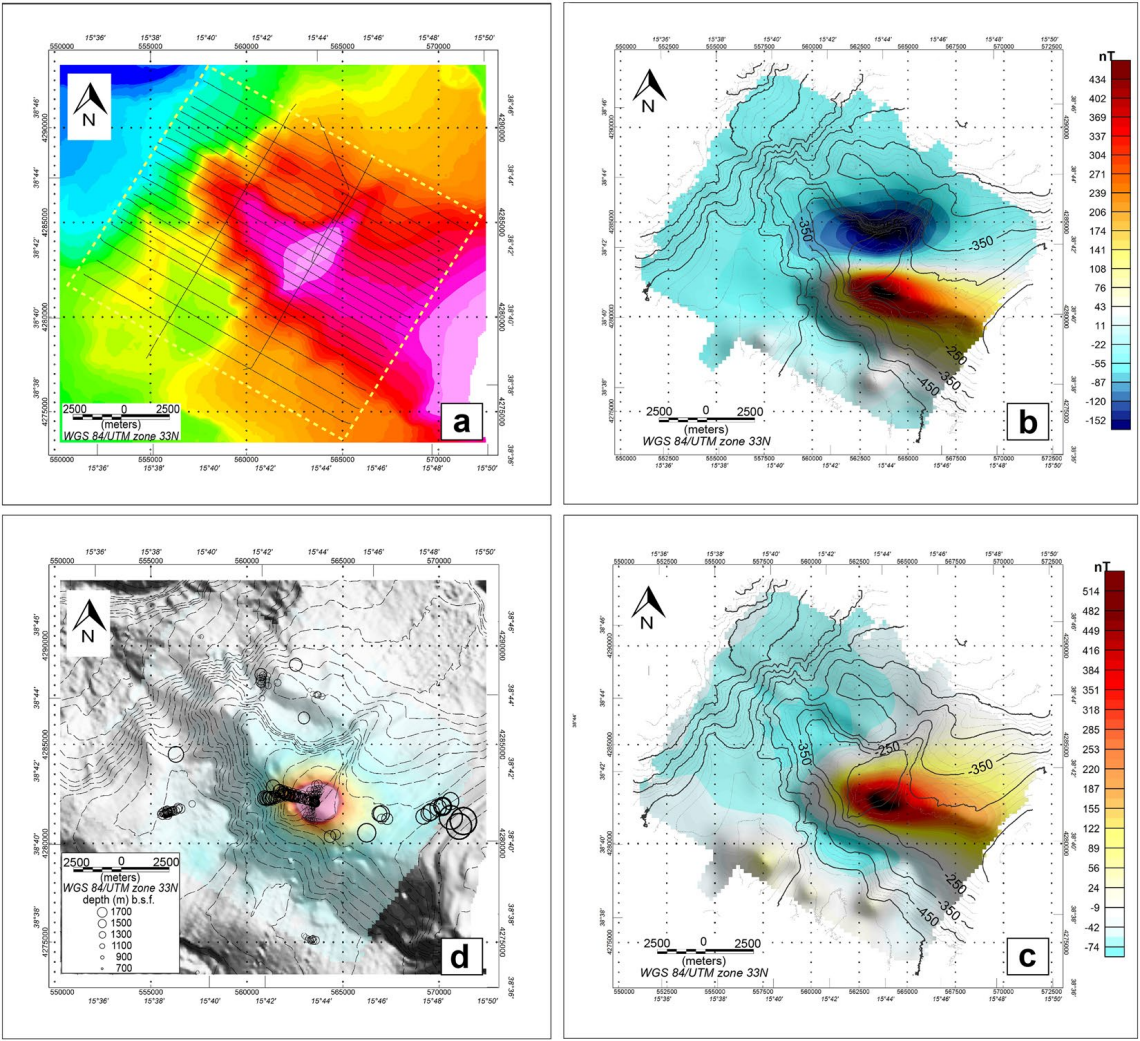
Shipborne magnetic data. (**a**) Locations of high resolution magnetic profiles (thin black lines), collected during the GEOCAL-14 cruise, over-imposed to the bathymetry. (**b**) Map of the magnetic anomaly field. Bathymetric contours (thin black lines) with 100 m step are over-imposed. (**c**) Map of the reduced to the pole (RTP) magnetic anomaly. (**d**) Magnetic analytic signal and Euler’s deconvolution solutions (circles) over-imposed on gray-shaded CVR morpho-bathymetry. Circle size increases with the Euler’s solution depth below seafloor (b.s.f.). All the maps were obtained with the GMT package^80^.

Magnetic investigations across Capo Vaticano and neighboring areas aimed at recovering details of the magnetic anomaly pattern related to the high magnetized body suggested by previous studies below the faults-controlled structural-high forming Capo Vaticano Ridge. Indeed, the new shipborne data provide high resolution magnetic anomalies over the CVR (Fig. 5a) and add further details to the aeromagnetic data discussed in ref. ^27^.

The CVR structure displays high amplitude magnetic anomalies ranging from −169 to 468 nT, mostly oriented E-W (Fig. 5b). The dipole is clearly centered on the morphologic high with low-frequency low-amplitude anomalies in the surrounding area suggesting no other causative sources. The total intensity magnetic anomaly field was then reduced to the magnetic pole (RTP) to remove its dipolar behavior and to address a direct correlation between the maximum of magnetic anomaly and the center of the causative source (Fig. 5c). The RTP transformation was obtained by applying a phase shift in the FFT domain using the local values of inclination (54.6°) and declination (3.0°) of the Earth’s magnetic field as provided by the IGRF model^56^. RTP magnetic anomaly field ranges from −74 to 578 nT with the maximum centered exactly and lined-up with the summit of the structural high. The negative low-amplitude magnetic anomalies after the RTP correction, are probably related to a low-frequency contribution associated to the surrounding old and deep crustal portion. The shallowest portion of the CVR displays a strong positive RTP magnetic anomaly suggesting a shallow positively-magnetized body having the same polarity of the induced field. This allows us to define the lower bound of the timing of the emplacement of the magnetic source during the Brunhes chron (0.78–0 Ma), as previously proposed^25,26^.

Qualitative interpretation of the magnetic anomaly field was carried out by computing the three dimensional analytic signal (Fig. 5d). This technique integrates horizontal and vertical gradients of the magnetic signal in order to enhance the edges of the causative source^57,58^. The three-dimensional analytic signal results in a bell-shaped distribution having maximum amplitude along the lateral edges of the causative body. In the case of CVR, the analytic signal shows a clear circular pattern having its maximum positive value above the shallowest portion of the structural high affected by the NE-SW fault system, namely R1F^26^ (Figs. 2 and 5). This seems to indicate a main causative source or a cluster of sources just below the structural high without any evidence of widespread lateral additional magnetized material.

A quantitative analysis of the magnetic data provides an estimate of the average depth of the causative bodies. The depth distribution of the centroids of the magnetic sources (Fig. 5d) was computed using the Euler’s deconvolution technique^59,60^, based on a least-squares inversion of the Euler’s homogeneity equation for magnetic anomaly data^61^, adopting a structural index (SI) equal to 1 (tabular structures, sill and other intrusive features) because it provided the highest clustering of solutions. Calculations were carried out testing different values of SI (from 0 to 3) being related to the geometry of the source. The Euler’s deconvolution was applied interactively using fixed-size dynamic windows covering the entire magnetic anomaly grid. Distribution of Euler’s solutions ranges from 700 to 1700 m below the seafloor suggesting very shallow main causative bodies (Fig. 5d). Two main magnetic sources (or ensemble of sources) are clearly observed: (1) a very shallow magnetized body located at depths between 700 to 1000 m, aligned NNE-SSW and following the main tectonic pattern and the distribution of exhalation centres; (2) an E-W oriented deeper cluster of sources having an average centroids depth between 1400–1500 m with a clear deepening trend toward WSW (Fig. 5d).

### Hydrothermal flow numerical modelling

**Figure 6:**
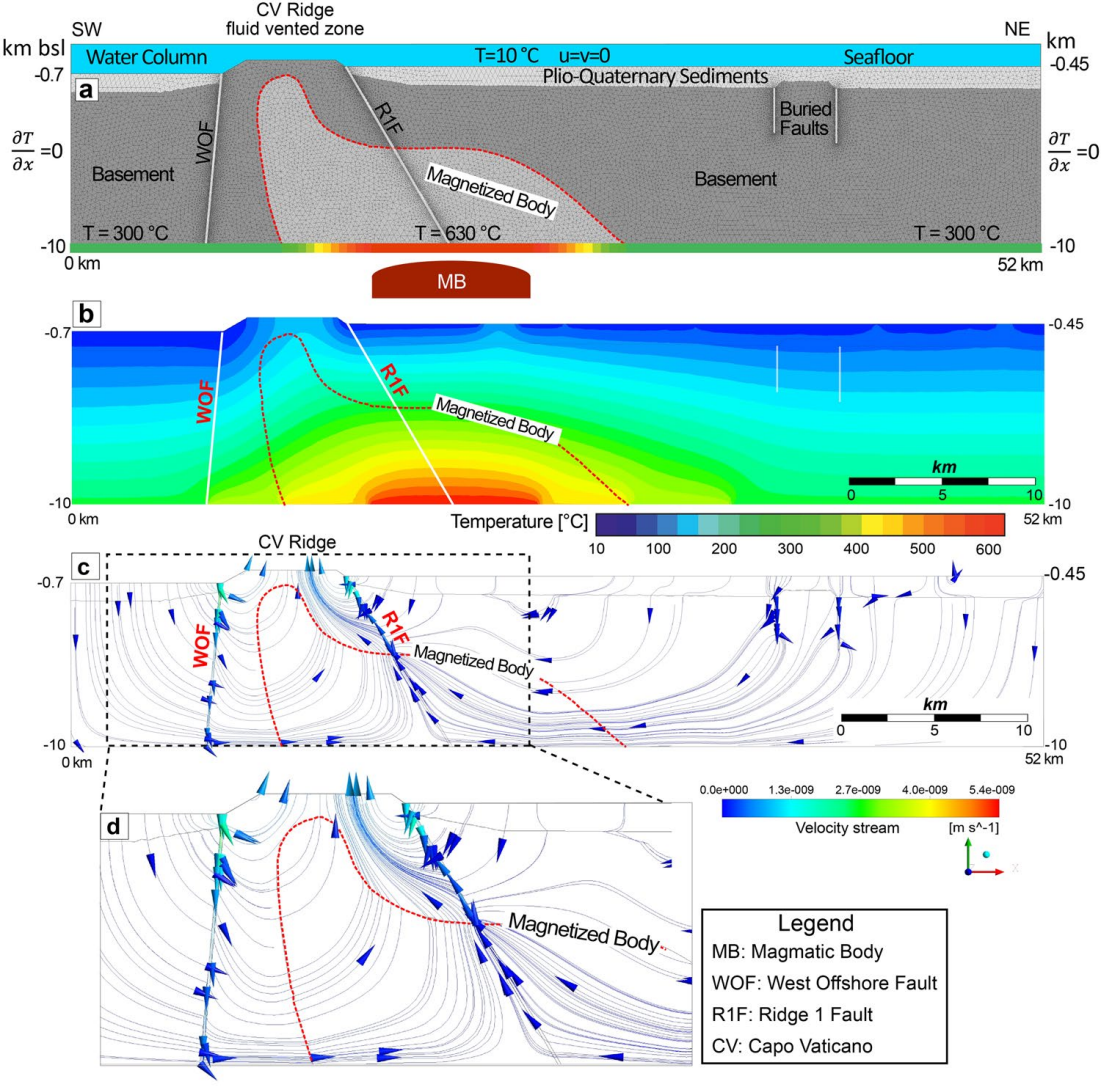
Hydrothermal flow numerical model. (**a**) Model box, triangular mesh and boundary conditions. White solid lines indicate faults. Red dashed line bounds the magnetized region below the CVR as inferred by magnetic modelling^26^. For numerical stability purpose, temperatures at the base of the model scale linearly from 300 °C to 630 °C on both side of the heat source assumed below the R1F and due to a deeper cooling magmatic body (MB). (**b**) Predicted temperature distribution. (**c**) Predicted flow pattern. Blue lines indicate the seawater particle streamlines and arrows the flow direction. Dashed box indicates the region displayed in (**d**) showing flow-pattern details. (**d**) Details of the predicted flow pattern below the CVR and along its bounding faults (WOF and R1F).

Following the geological interpretation of the seismic line GSE_06, a model box including the Plio-Quaternary sedimentary sequence, the pre-Messinian acoustic basement and the two structural highs bounded by two couples of normal faults was built. Faults were simulated as regions of higher permeability, with a constant width of 50 m (Fig. 6a). The seafloor was considered to be a permeable barrier^61^. Finally, a magnetized body as inferred from magnetic data inversion was included into the model by a region of lower permeability and porosity when compared with the surrounding units. The model takes also into account differences in sediment thickness and in the water column as observed in the seismic image (Fig. 3). This information was used to define the initial boundary pressure conditions at the top of the model.

There are some dominant aspects that determine the temperature distribution and the fluid flow pattern within the system: fault patterns, water column and sediment thickness variations, the presence of a magnetized body and of a deep-seated magmatic body below the base of the model. Previous magnetic inversion, based on aeromagnetic data, constrains the location and the temperature of the heat source^26^. The magnetized body is elongated E-W with the shallowest portion centered just below the CVR, while at greater depth, up to ~6–7 km, it becomes larger eastward and northward with the northern boundary about parallel to the R1F strike^25^. The magnetized body requires a temperature well below the Curie temperature for magnetite (585 °C). This suggests a cooling source centered below the R1 fault with a temperature >585 °C and deeper than the modelled magnetized body (Fig. 6a). Next, we discuss the effects of model geometry and parameters on fluid flow and temperature distribution separately.

#### Effects on temperatures and fluid flow of faults and pressure differences

The effects of faults permeability and water column pressure variations on hydrothermal flow were investigated focusing on faults that bound the CVR. These faults are interpreted as active pathways for fluid flow because in the seismic sections they cut the entire sedimentary sequence up to the seafloor (Figs. 3 and 4). Although in the profile GSE_06, these faults are clearly identified up to a depth of 1.5 km, in our preferred numerical model (Fig. 6) we extended them up to a depth of 10 km according to the interpretation that WO and R1 faults determine and control the geometry of the structural high of the CVR. In addition, we assumed fault’s dip angles as observed in the seismic section projected perpendicular to the fault’s planes, i.e., 85° and 60° for WOF and R1F, respectively. However, in order to evaluate how fault-dip and maximum depth may affect the deep hydrothermal flow pattern, we carried out numerical experiments adopting vertical faults (Supplementary Fig. 1) and a fault extension with depth of 1.5 km (Supplementary Fig. 2). Although fluid flow velocities and flowlines are different, all models predict a convective flow below CVR with fluid escapes at its summit.

The calculated isotherms are smooth and the temperature gradient away from the CVR and the faults (Fig. 6b) is close to the initial conductive condition. Below the CVR, the resulting isotherms run sub-parallel to the faulted flanks with a temperature gradient that decreases toward the SW flank due to a predicted downward flow along the WO fault plane. On the NE flank, uprising fluid flow determines an elevated thermal gradient with a shallow circulation pattern along the R1 fault plane and flux pathways emerging at the seafloor on the summit of the CVR (Fig. 5) with relatively low flow rates (3.2232 × 10^−9^ m/s) and where the estimated temperatures on outlet vents vary between 10 to 100 °C, with an average temperature of 53 °C. Porous flow in a non-deformable matrix is driven mostly by rock permeability distribution (Fig. 6 and Supplementary Figs. 3 and 4) and buoyancy forces (Fig. 6 and Supplementary Fig. 5). Although the two faults in the model are simulated with the same physical parameters, they affect differently the fluid flow pattern and the temperature distribution, mostly due to convective flow determined by the heat source location. The modelled shallow flow pattern is dominated by permeability distribution, thus WOF, overlain by a Plio-Quaternary sedimentary sequence thicker than that overlying R1F, shows a downward flow of cold seawater through the shallowest part of the model with higher velocities (Fig. 6c,d). Downward fluid flow is also predicted through the R1 fault plane down to 2.5 km from the seafloor with velocities higher along the fault than across the basement due to the higher fault permeability. In the deepest part of the model below the CVR flow paths are deflected toward the R1F and deep upward flow along the R1 fault plane is predicted. This is mostly due to the higher temperatures in the central part of the bottom of the model accounting for the assumed deep magmatic body centered below R1F. Away from the CVR and its bounding faults, low flow velocities are predicted (2 × 10^−11^ m/s) through the entire sedimentary sequence (Fig. 6c). The buried structural high to the NE of the section does not affect much fluid flow patterns given the resulting low velocities similar to those of the sediments above.

#### The effects of the magnetized body on temperatures and fluid flow

The magnetized body is simulated with a different lithological unit characterized by porosity and permeability lower than the hosting rocks. The magmatic heat source is assumed to be 12 to 28 km deep^25^, while the associated plumbing system, originating the shallow magmatic intrusions up to ~1 km below the CVR, represents the observed magnetized body that dips toward NE^26^ with an angle of ~45°. Several simulations were carried out with permeability of the magnetized body ranging between 0.1 × 10^−17^ and 0.5 × 10^−17^ m^2^. The results do not show any significant change in fluid flow pattern but only slight changes in flow velocities and temperature distribution. However, the numerical model predicts fluid flow pathways beneath the CVR away from the magnetized body in the shallowest part of the model (Fig. 6c,d).

## Discussion

### Structural model and fluid flow pattern

**Figure 7:**
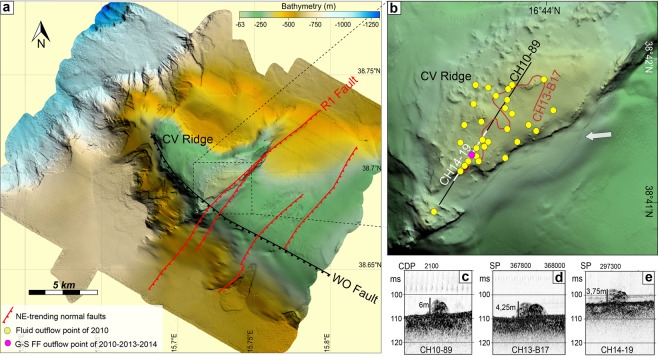
Morphology of the CVR summit. (**a**) Shaded relief images of the Capo Vaticano Ridge from a 5 m cell-size bathymetric grid. The rough topography visible in places in the vicinity of the R1 fault is due to rocky blocks (by ROV inspections), probably erosional relicts formed during the last sea level rise. The black dashed box outlines the R1F morphological details shown in (**b**). (**b**) Locations of fluid vents (filled yellow circles) and high seismic profiles crossing the vent area at the top of the CVR. (**c**) CH10-89, (**d**) CH13-B17 and (**e**) CH14-19 sub-bottom Chirp profiles acquired during the 2010, 2013 and 2014 surveys, respectively. Chirp profiles show long lasting vent-related water velocity anomalies in the proximity of seafloor.

Seismic data define the tectonic setting of the structural high forming the CVR. The depth-migrated profile GSE_06 (Fig. 3) is crucial in defining the geometry of the structural high and of the surrounding sedimentary basins furnishing the base for the geological section used in the numerical fluid-flow modeling, whereas sparker profiles and high resolution swath bathymetry highlight surficial fault patterns and present day activity of R1 fault (Figs. 4 and 7). The Pleistocene WOF and the Holocene R1F, and the location of heat-source control the thermal convective flow pattern below the CVR where a deep hydrothermal circulation pattern is predicted that may reach the depth of 10 km with the main faults serving as main pathways for fluid flow. In our model, the calculated Rayleigh-Darcy number of 4.6 is well below the critical value (17.65) required for convective flow in an open top system^62^. However, these two large faults representing important crustal discontinuities are able to trigger convection below the CVR, as already suggested by other authors in similar tectonic setting^63,64^, favored by the presence at depth of a low permeability region (the magnetized body). Our results show that the combined effects of seafloor morphology, sediments thickness, permeability distribution and of a deep magmatic cooling body can reverse the fluid-flow pattern from downward to upward along the fault planes of the two major faults. Heat source centered below the R1F induces an upward flow along the R1 fault plane that, interacting with the shallow downward circulation, forces the fluid to escape at the summit of CVR. Also the presence of a shallow magnetized body just below the CVR changes the fluid circulation path and plays an important role in promoting fluid escape at the summit of the structural high (Fig. 6). Model results are in agreement with observations. In fact, Chirp profiles acquired during the three surveys carried out in the study area, reveal a region of fluid vents confined at the summit of the CVR focused mostly in an area closer to R1 fault (Fig. 7a,b). The relatively low rate of fluid outflow estimated from the model is in agreement with the decreasing plume-height of major vents through time observed between 2010 and 2014 (Fig. 7b,c,e), and with the presence of several minor vents detected only in 2010. Vent-location spatial variability and vent-intensity temporal variations may be explained by a weak source that need time to recharge the entire system^28^. In addition, numerical modelling suggests high permeability zones along both fault planes despite their different chronostratigraphic age (Fig. 6).

The region of high temperature at the model bottom, simulating a deep seated cooling magmatic body (>10 km), is required in order to reverse the downward flow and to allow fluid escapes at the summit of the CVR (Fig. 6). Seawater entering within the upper crust through the main faults may interact and trigger ionic exchange with both magmatic rocks and fluids of volcanic origin. This interaction may explain the observed enrichment in δ^3^He relative to δ^4^He of the discharged fluids, a feature frequently observed at submarine volcanoes of the Eolian arc^65–67^ or in oceanic island systems^68^. Modeling results are in agreement with the values of δ^3^He measured at CVR vents^28^ ranging from 5.0 to 9.68%.

High resolution shipborne magnetic anomalies (Fig. 5c) confirm the presence of a highly magnetized region (>500 nT) below the central part of the CVR and across the R1F reaching a minimum depth of ~700 m, as inferred by Euler’s deconvolution results (Fig. 5d). This refines previous interpretations based on aeromagnetic data^26^. Furthermore, the shipborne data show a complex trend of the shallowest part of the magnetized region, with a top surface not regular or perfectly horizontal contrary to what was suggested previously. In fact, Euler’s deconvolution (Fig. 5d) reveals that the shallowest portion (depths from 700 to 900 m) of the magnetized body dips NNE, while the portion with depths from 1000 to 1500 m dips WNW. Some scattered Euler’s solutions deeper than 1500 m are present closer to the CV promontory, suggesting a magnetic body that bends at depth toward ESE, as inferred by aeromagnetic data inversion^25^. The complexity added by the new magnetic data suggests that the highly magnetized region is probably due to magmatic intrusions, such as sills and dykes, whose ascent has been partially controlled by the two main faults bounding the structural high. Contrary to what suggested in previous works^26,27^, the ascending magma has never reached the seafloor as suggested by Euler’s solution depths (Fig. 5d), and by the absence of recovered volcanic rocks and of volcanic morphologies at the summit and along the flanks of the CVR (Fig. 7).

**Figure 8:**
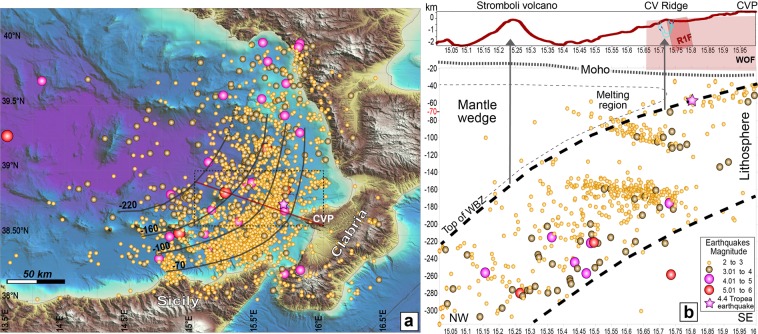
Earthquake depth distribution below the southern Tyrrhenian Sea. (**a**) Shaded relief map of the SE Tyrrhenian Sea showing instrumental seismicity recorded from 2000 to 2018 (http://cnt.rm.ingv.it/search). Earthquake epicenters are indicated by colored circles with colors indicating the magnitude of events, see legend in (**b**). Bathymetry data from EMODnet (*EMODnet Bathymetry Consortium*, *2016: EMODnet Digital Bathymetry*, http://doi.org/10.12770/c7b53704-999d-4721-b1a3-04ec60c87238) and elevation data from SRTM30 grids (*David T*. *Sandwell*, *Walter H*. *F*. *Smith*, *and Joseph J*. *Becker*. *The Regents of the University of California*. *All Rights Reserved*). Dark grey solid lines indicate the depth of the top of Wadati Benioff Zone (WBZ); red solid line represents the depth-section location shown in (**b**) CVP: Capo Vaticano Promontory. (**b**) Depth section from the eastern margin of Calabria to Stromboli crossing the Capo Vaticano Ridge. Grey arrows represent possible pathways for melt migration from the region above the WBZ where partial melting of the mantle wedge may occur.

The δ^3^He/δ^4^He anomalies in the water column suggest a very deep source, related to mantle-wedge partial melting when compared with contents of volcanic gases from subduction setting^69^. Generally, the depth to which a subducting slab is capable to promote partial melting of the mantle-wedge with an associated volcanic arc ranges from 86 to 160 km^37,70,71^. More recently, a global compilation of slab depth variations beneath volcanic arcs extended this range from 72 to 173 km^72^. Following the method of ref. ^73^, we depicted the top of the Wadati Benioff Zone (WBZ) mapping at depth earthquake hypocenters recorded from 2000 to 2018 in the southeast Tyrrhenian basin (Fig. 8a). Focal zone locations of earthquakes with magnitude ranging from 2 to 6 (M_w_) describe well the Ionian subducting lithosphere below Calabria and the Tyrrhenian sea. Accordingly, the top of the WBZ below the CVR has been estimated to be ~70 km deep (Fig. 8b), in agreement with the focal depth of the event with M_w_ 4.4 occurred offshore Capo Vaticano on July 14^th^, 2018 (Fig. 8). Figure 8b shows a depth section from Capo Vaticano to Stromboli including earthquake hypocenters, top and bottom of WBZ, Moho depth^35,74^, crustal tectonic features and fluid circulation pattern. The WOF likely reaches the Moho discontinuity at the estimated depth of 22 km.

Temporal evolution of geochemistry of erupted lavas from the Aeolian arc suggests that Ionian slab subduction is presently ended^75^. In addition, geodetic measurements indicate that also subduction-plate roll-back is almost halted^76–78^ with the western Sicily that moves together with the Nubia Plate. Instead the Calabrian inner arc, decoupled from the subduction slab, is uplifting at an average rate of ~1.6 mm/a at Capo Vaticano^79^. This implies a reduction in down-welling mantle flow above the WBZ and an increasing distance of the overriding plate from the WBZ with a consequent rising of mantle-wedge isotherms. Thus, we suggest that the magmatic intrusions below the CVR are related to melt migration from a deep source due to partial melting of mantle-wedge favored by subdued sediments dehydration at the top of the WBZ. We suggest that WO and R1 fault-planes act as preferred pathways for melt ascent through the crust. However, the magmatic system below the CVR cannot be linked to that giving rise to the Aeolian arc, because it is located in a much inner area. It may be related to a more recent and shallower partial melting region above the WBZ due to decoupling between the Calabrian arc and the subduction slab, extending the southern Tyrrhenian arc-related magmatic system toward the inner arc (Calabria-Peloritani).

## Methods

### Geophysical data

Three geological-geophysical surveys (ISTEGE, ISTEGE-2, and GEOCAL-2014) were carried out offshore western Calabria between 2010 and 2014, where we collected a multiscale and multidisciplinary dataset composed by:

- 2231 km^2^ of swath bathymetry data (MB1) covering the entire S. Eufemia Gulf. More than 800 km^2^ of high resolution multibeam (MB2) and shipborne magnetic data covering the shallowest part of the CVR (Fig. 2);
- 330 km of multichannel seismic reflection (MCS) profiles (Figs. 2 and 3), 2223 km of high resolution sub-bottom Chirp profiles and about 30 km of single channel sparker profiles (Fig. 2);
- Water samples by using 24-Niskin bottle Rosette and 4 HD videos recorded by ROV have been collected along the summit of the CVR; in addition to four dredging stations located on the eastern flank of the CVR where only sediments were recovered.

MB1 data were acquired using software PDS2000 by two hull mounted multibeam systems: a RESON Seabat 8111 (110 kHz) and a Seabat 8150 (12 kHz), working at two different water depth ranges that adequately combined allow to cover depth >20 m. MB2 data were acquired with package Kongsberg SIS using the Kongsberg-Maritime EM710 multibeam system (70 kHz) operating in a water depth range of 3–1200 m. The two datasets were processed in order to remove noise due to errors of the navigation and acquisition systems, and then combined to produce a regional scale map with a 20 × 20 m grid cell size and a local scale map with 5 × 5 m grid cell size. Spatial analysis and mapping were performed using the GMT package^80^.

Single channel sparker profiles, with sample interval of 0.25 ms and total record length of 2 s, were processed using the commercial software Focus/Disco by Paradigm in order to remove incoherent noise and improve primary energy. Processing sequence consisted of: trace editing; resampling; trace amplitude equalization and spherical divergence correction in order to recover weaker signals at depth; f-x deconvolution in order to improve lateral continuity of shallow sedimentary layers; trace mixing to improve lateral continuity and decrease random noise; time variant band pass filter; constant velocity time migration by a finite difference method adopting the water velocity (i.e., 1500 m/s).

### Pre-stack depth migration

Profile GSE_06 (Fig. 3) from the MCS dataset has been selected for Pre-Stack Depth Migration (PSDM) in order to better define geometry of reflections and faults pattern. The resulting geological section was used as basis for the fluid-flow numerical modeling. Processing was performed with GeoDepth software by Paradigm applying iteratively the PSDM (based on the Kirchhoff algorithm) and adopting at each iteration a velocity grid obtained with a tomographic method. This procedure allows to define a detailed velocity model and reliable depth information. The method uses the PSDM output, represented by the Common Image Gathers (CIGs), to determine iteratively an accurate velocity field^81^. The first iteration is performed by using a constant velocity model (i.e., the water velocity) that allows flattening of seafloor reflections. Then, an updated velocity grid is created by using interval velocities derived from the root-mean square (RMS) velocities obtained during the conventional processing. Finally, combining seafloor reflection picking with the updated velocity field, an initial velocity model is created including constant water velocity above the seafloor and interval velocities below. The resulting velocity grid is then used to create the first PSDM section. A residual depth move-out analysis is carried out at every gathers in order to create additional interlayers. The velocity field is then updated with these two datasets as input for tomography. Iteration is repeated until all picked horizons are flattened. The final PSDM is obtained by using the last velocity grid.

### Magnetic data

Shipborne magnetic data were collected with R/V Urania during cruise GEOCAL-2014, using a SeaSpy marine magnetometer towed about 180 m astern. Total magnetic field data were sampled at 1 Hz by using the specific SeaLink software suite that also provides a synchronous DGPS layback-corrected position for the tow-fish. The magnetic survey (Fig. 5a) was conducted following a set of 21 track lines oriented NW-SE and spaced 500 m apart with the outermost profiles spaced 1000 m. Two additional NE-SW control tie-lines, spaced 5000 m, were also surveyed to acquire a set of cross-over points useful for following data processing. The resulting dataset counts about more than 103400 observation points collected along 380 km of magnetic lines. Raw magnetic data were corrected removing spikes and outliers. An additional heading correction was required in order to minimize the directional magnetic contribution of the vessel mass^82^. Diurnal variations of the Earth magnetic field were computed using the base station data from Duronia observatory, Italy (belonging to the InterMagnet observatories); then corrected data were levelled and smoothed applying statistical analysis of cross-over error matrix. Finally, magnetic anomalies were calculated by subtracting the IGRF (International Geomagnetic Reference field) model^56^ from the observed Earth’s magnetic data.

### Fluid flow numerical modelling

We used two-dimensional numerical simulations of coupled fluid flow and heat transport based on the finite volume method. The commercial computational fluid dynamics software ANSYS Fluent was used to calculate the models (ANSYS User Guide, 2011). Darcy’s law is assumed to hold and the fluid has uniform thermodynamic properties (i.e., thermal expansion coefficient is constant), while the density decreases linearly with temperature obeying to the Boussinesq approximation.

#### Initial and boundary conditions

The model box, with sizes of 52 × 10 km and based on the geological section derived from the seismic depth section GSE_06 (Fig. 3), has been discretized by a uniform triangular mesh with an area of the faces ranging from 5 m to 250 m depending on faults distance. 70533 grid nodes and 34992 triangular elements satisfying the Delaunay criterion were used in the calculation. Each litho-stratigraphic unit has been assumed non-deformable (neglecting compaction), and isotropic and homogeneous respect with its physical properties, i.e., permeability, porosity, heat capacity and thermal conductivity (Supplementary Table 1). For modeling purposes, we have considered only two main units: the pre-Messinian acoustic basement and the Plio-Quaternary sedimentary unit (PQSU). Previous studies on individual hydrothermal systems have shown that permeability variations by one order of magnitude between lithological units represent a good approximation in numerical modelling^18,19,62,83^. In our preferred model we assumed permeability values of 1 × 10^−15^ m^2^, 1 × 10^−16^ and 1 × 10^−17^ m^2^, for faults, sediments and basement, respectively. In order to explore how permeability affects flow pattern in our numerical models we carried out experiments assuming different permeability values (Fig. 6 and Supplementary Figs. 3 and 4). An open-top rectangular model box was created to carry out the simulations. At the top of water and sediments/rocks interface we assumed an initial pressure that depends on the water column height. This pressure generates a vertical flow with fluid being free to enter into geological units at constant temperature (seafloor temperature of 10 °C) and leave the system with zero cooling rate (dT/dz = 0). At the bottom of the model we assumed fixed temperature boundary conditions (300 °C) outside a central region below the CVR and part of S. Eufemia Gulf, where we assumed a temperature of 630 °C due to heat produced by a deeper cooling magmatic body. We assumed a linear temperature change from 300 °C to 630 °C at the transition between hosting rocks and the heat source at the base of the model to increase numerical stability (Fig. 6a,b). Below the CVR, in the central part of the model, the magnetized body (assumed due to solidified and cold magmatic intrusions) was simulated by a region of reduced permeability^84^. The vertical sides of the model were assumed to be impermeable, resulting in no fluid or heat flow transfer across the boundaries (Fig. 6).

## Supplementary information

Supplementary Information
