# Supplementary material for: Fault-controlled deep hydrothermal flow in a back-arc tectonic setting, SE Tyrrhenian Sea
Source: Sci Rep. 2019 Nov 27;9:17724. doi: 10.1038/s41598-019-53696-z (PMC6881442; doi:10.1038/s41598-019-53696-z)
Supplement: Supplementary file 1 — Supplementary Information [file 41598_2019_53696_MOESM1_ESM.docx]

## Supplementary Information

Fault-controlled deep hydrothermal flow in a back-arc tectonic setting, SE Tyrrhenian Sea

Maria Filomena Loreto^1^, D. Düşünür-Doğan^2^, S. Üner^2^, Y. İşcan-Alp^3^, Ocakoğlu N.^2^, Luca Cocchi^4^, Filippo Muccini^4^, Patrizia Giordano^1^, Marco Ligi^1^

^1^ *CNR - National Research Council of Italy, ISMAR - Marine Sciences Institute in Via Gobetti 101, 40129 Bologna, Italy.*

^2^ Department of Geophysical Engineering, Faculty of Mines, Istanbul Technical University, Maslak, 34469 Istanbul, Turkey.

^3^ Department of Geophysics, Faculty of Engineering, Istanbul University, Avcılar, 34850 Istanbul, Turkey.

^4^ Istituto Nazionale di Geofisica e Vulcanologia, Via di Vigna Murata 605, 00143 Roma, Italy.

**This PDF file includes:**

Supplementary Table 1

Supplementary Figures 1 to 9

References

* To whom correspondence should be addressed. E-mail: *filomena.loreto@bo.ismar.cnr.it*

| Parameter | Value | Unit |
| --- | --- | --- |
| Fluid density (Boussinesq) | 1000 | kg/m^3^ |
| Fluid dynamic viscosity | $\eta$*(T)* | kg/m s |
| Fluid specific heat capacity | 4200 | J/kg K |
| Fluid thermal conductivity | *k_f_(T)* | W/m K |
| Fluid thermal expansion coeff. | 2.07e^-4^ | 1/K |
| Gravity acceleration | 9.810 | m/s^2^ |

| Unit | Density  (kg/m^3^) | Permeability  (m^2^) | Thermal conductivity  (W/m K) | Specific heat capacity (J/kg K) |
| --- | --- | --- | --- | --- |
| WOF | 3100 | 1.0e-15 | 2.5 | 840 |
| R1F | 3100 | 1.0e-15 | 2.5 | 840 |
| Bedrock | 3100 | 1.0e-17 | 3.0 | 840 |
| Magnetic body | 3100 | 0.5e-17 | 2.5 | 840 |
| Buried faults | 3100 | 1.0e-15 | 1.5 | 840 |
| Sediments | 1800 | 1.0e-16 | 1.5 | 1500 |

**Supplementary Table 1 | Model physical parameters for fluid and geological units.** Dynamic viscosity $\left( \eta\right)$ and thermal conductivity (*k_f_*) of the fluid are assumed to vary with temperature according to refs. 84 and 85, respectively. The assumed functions result: $\eta\left( T \right)=\exp\left[ A+B/(C+T) \right]$*,* where *A =* 3.7188, *B =* 578.919 and *C =* -137.546; and $k_{f}(T)=-0.92247+2.8395 A-1.8007 A^{2}+0.52577 A^{3}-0.07344 A^{4}$, where *A = (T+*273.15*)/*273.15.


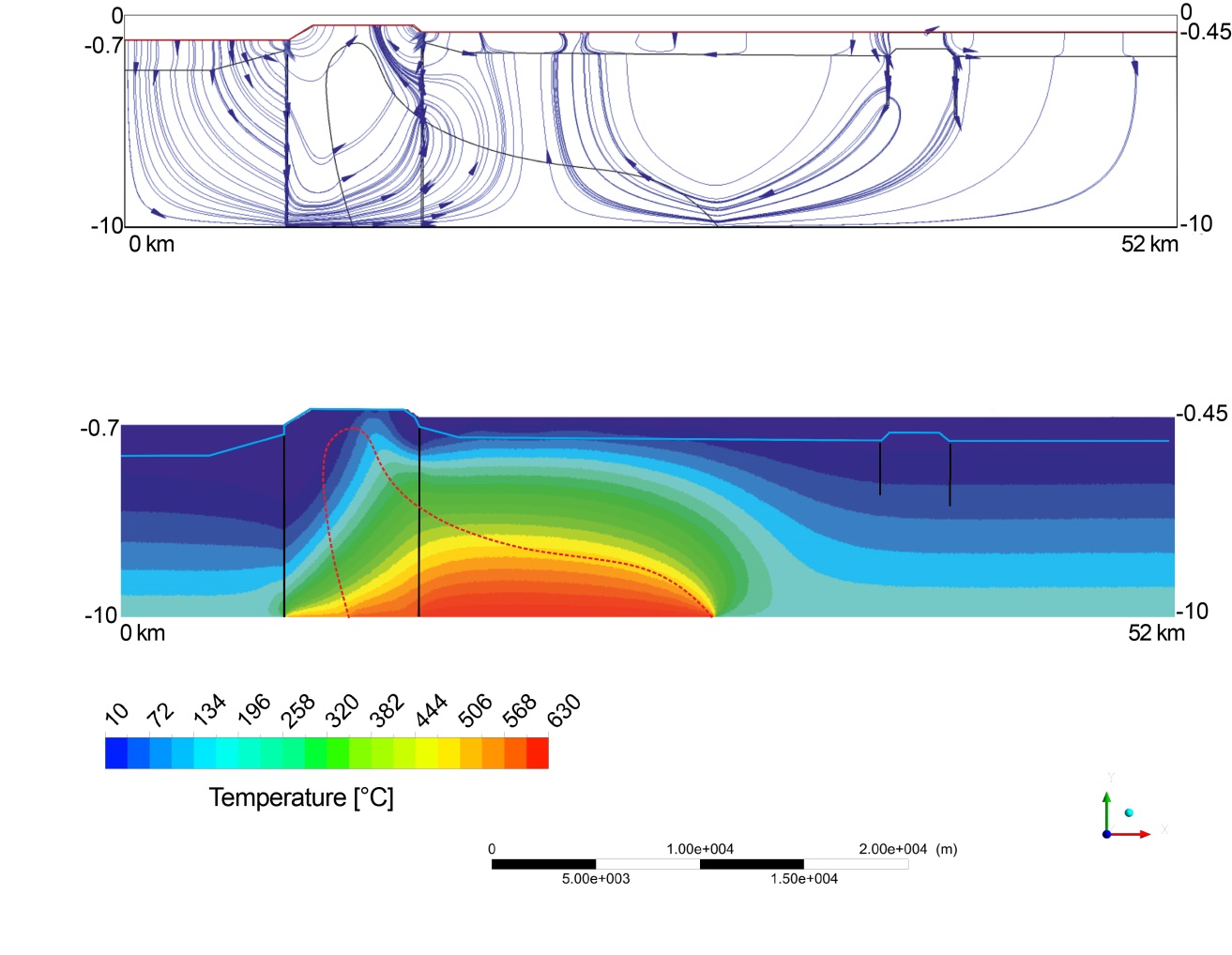


**Supplementary Figure 1 | Testing fault dip-angle in numerical flow model.** Fluid flow pathways (top) and temperature distribution (bottom). WO and R1 faults are assumed to extend to a depth of 10 km with a dip of 90°. Physical parameters of fluid and geological units are listed in Supplementary Table 1.


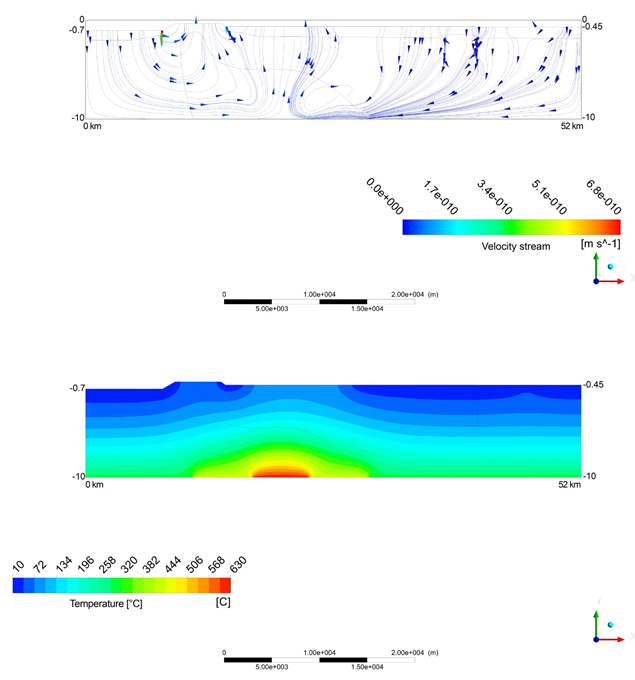


**Supplementary Figure 2 | Testing fault maximum depth in numerical flow model.** Fluid pathways and flow velocities (top), and temperature distribution (bottom). Faults bounding the CVR (WOF and R1F) are assumed to be 1.5 km deep. WOF is assumed sub-vertical dipping to the SW at an angle of 85°, while R1F dips to the NE at an angle of 60°. Model physical parameters as in Supplementary Table 1. Although WOF and R1F maximim depth is limited to a depth of 1.5 km, a convective flow is predicted below CVR driven by permeability distribution and heat source location. Instead, the returning flow below the S. Eufemia basin (right side of the section) is induced mostly by the assumed impermeable model bottom.


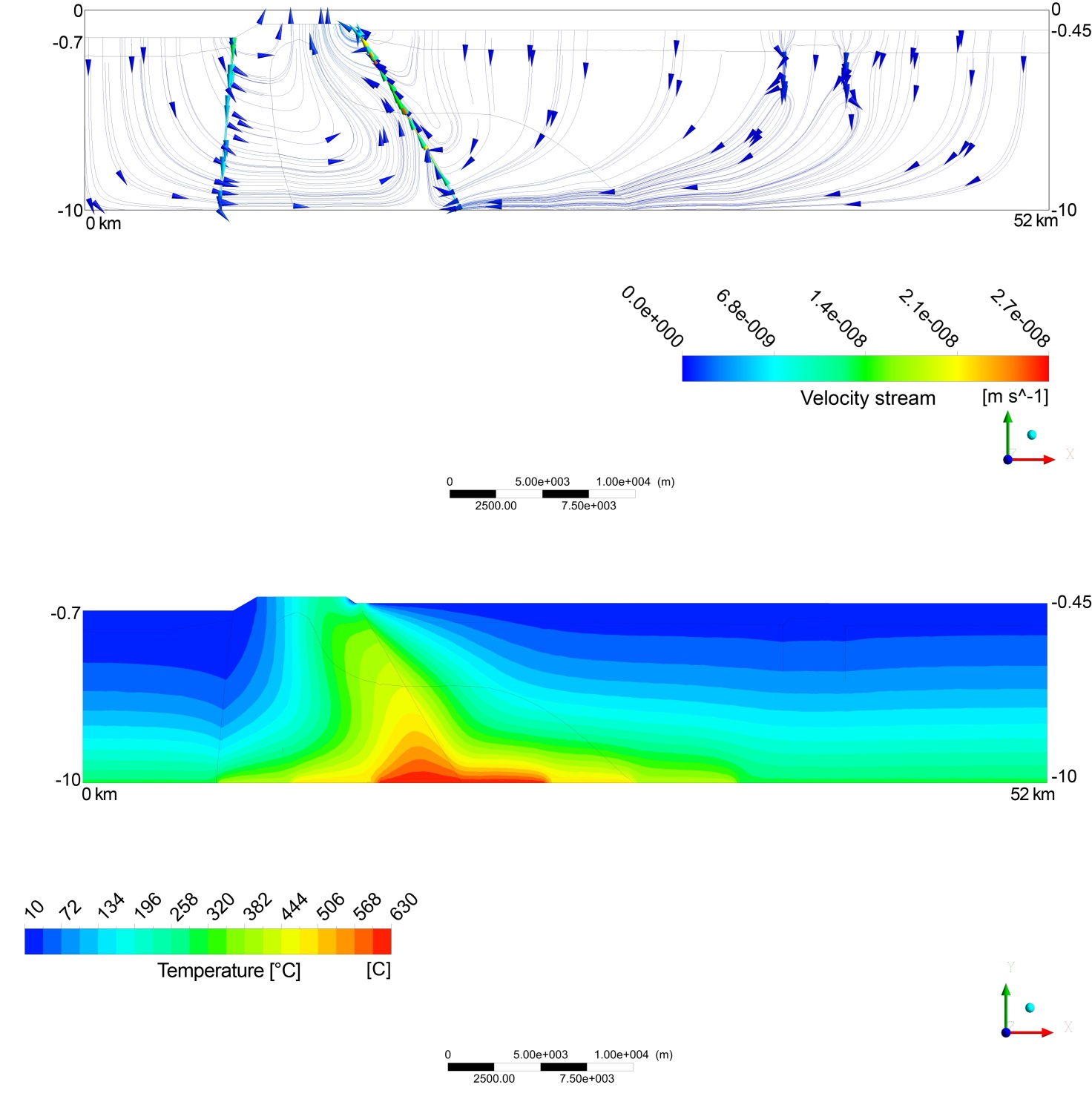


**Supplementary Figure 3 | Testing permeability distribution in numerical flow model.** Fluid pathways and flow velocities (top), and temperature distribution (bottom). Faults bounding the CVR are assumed to be 10 km deep. WOF is assumed to dip to the SW at an angle of 85°, while R1F dips to the NE at an angle of 60°. Fluid and basement physical parameters as in Supplementary Table 1. Assumed permeability for sediments and fault zones are 1.0^-15^ and 1.0^-14^ m^2^, respectively. Results show high flow velocities along the fault-planes and indicate R1F as the main conduit for hydrothermal circulation. Model does not converge to steady state and results are strongly time dependent.


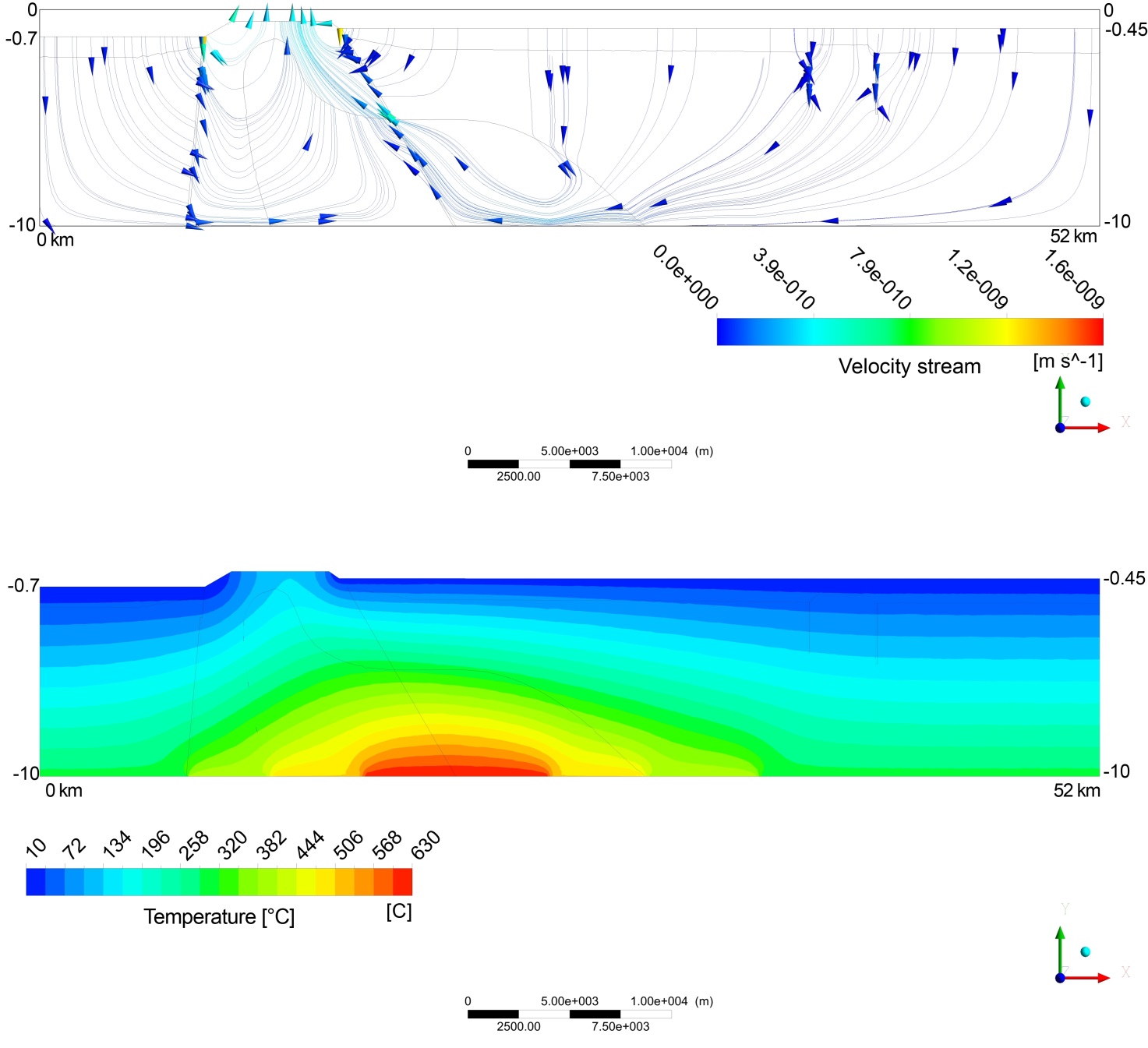


**Supplementary Figure 4.** **Testing permeability distribution in numerical flow model.** Fluid pathways and flow velocities (top), and temperature distribution (bottom). Faults geometry as in Supplementary Fig. 3. Assumed permeability for sediments and fault zones are 1e-17 and 1e-16 m^2^, respectively. The lower permeability of faults implies lower fluid-flow velocities and lower temperatures below the CVR. Model converges to steady state condition.


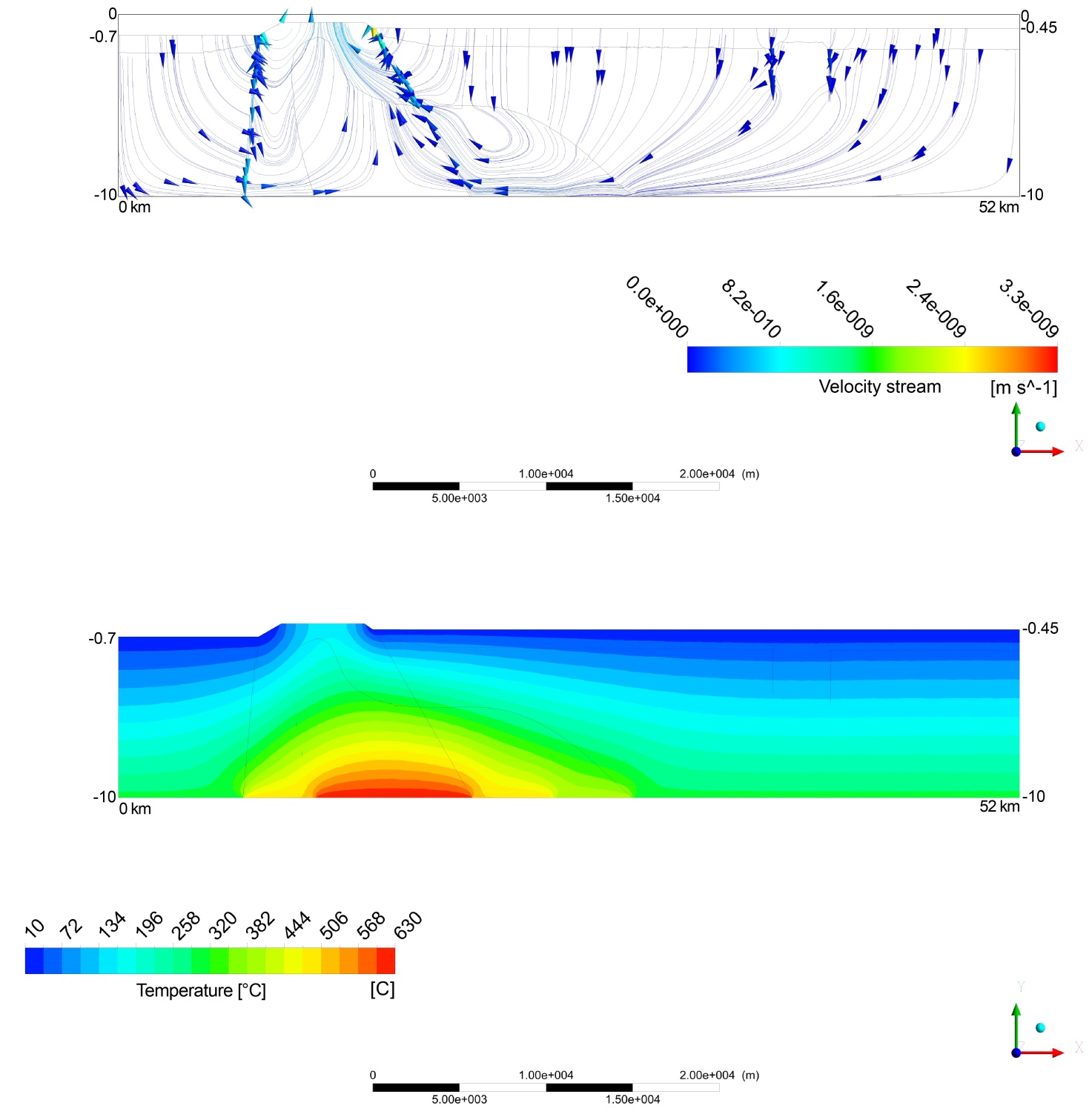


**Supplementary Figure 5 | Testing heat source location in numerical flow model.** Fluid pathways and flow velocities (top), and temperature distribution (bottom). Heat source location is moved toward WOF by 5 km. Faults geometry as in Supplementary Fig. 3. Fluid pathways and flow velocities (top), and temperature distribution (bottom). Physical parameters of fluid and geological units are shown in Supplementary Table 1.


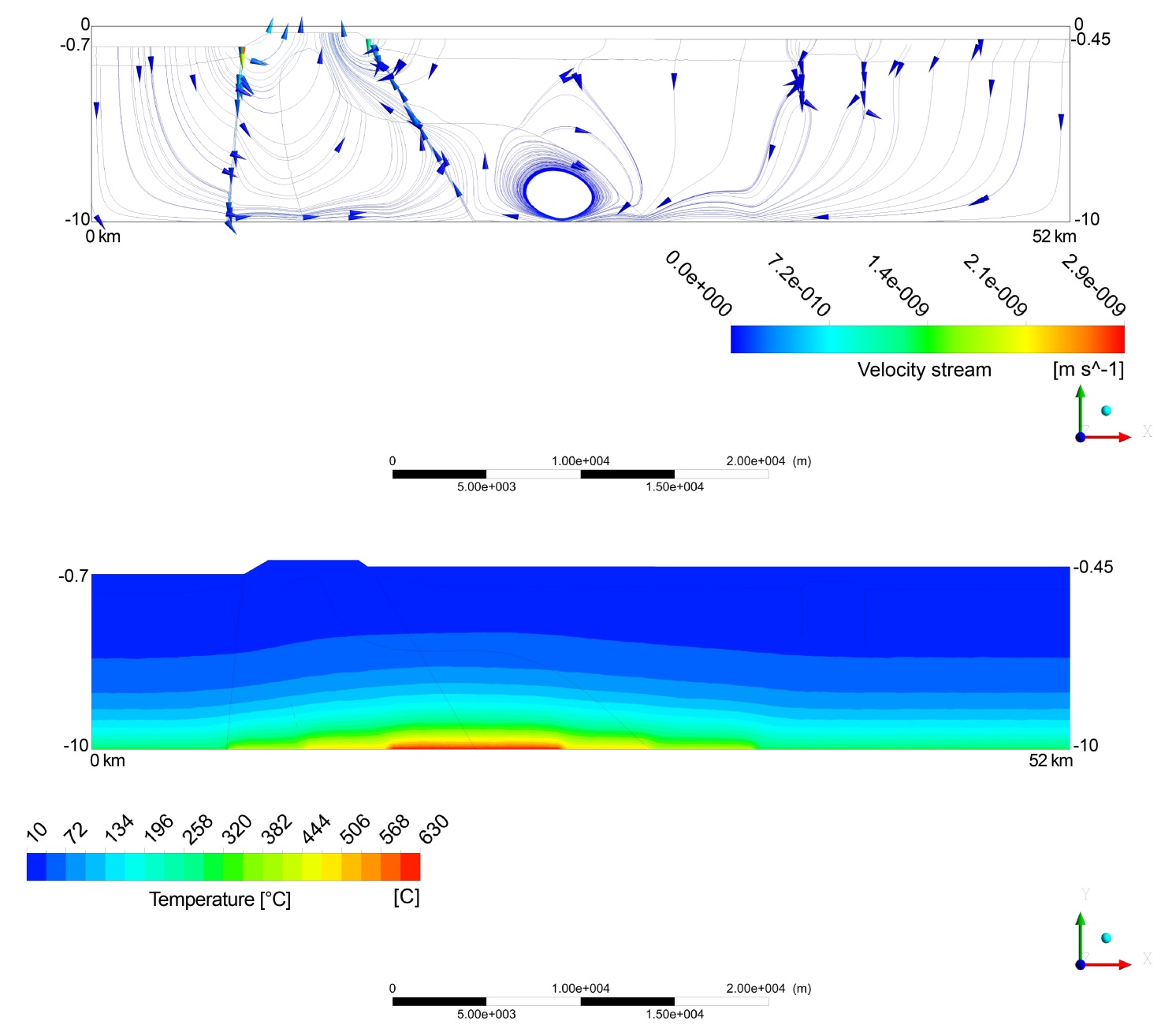


**Supplementary Figure 6 | Testing converge of numerical flow model solution.** Model snapshot at 100 ka. Fluid pathways and flow velocities (top), and temperature distribution (bottom). Model geometry and physical parameter as in Fig. 6.


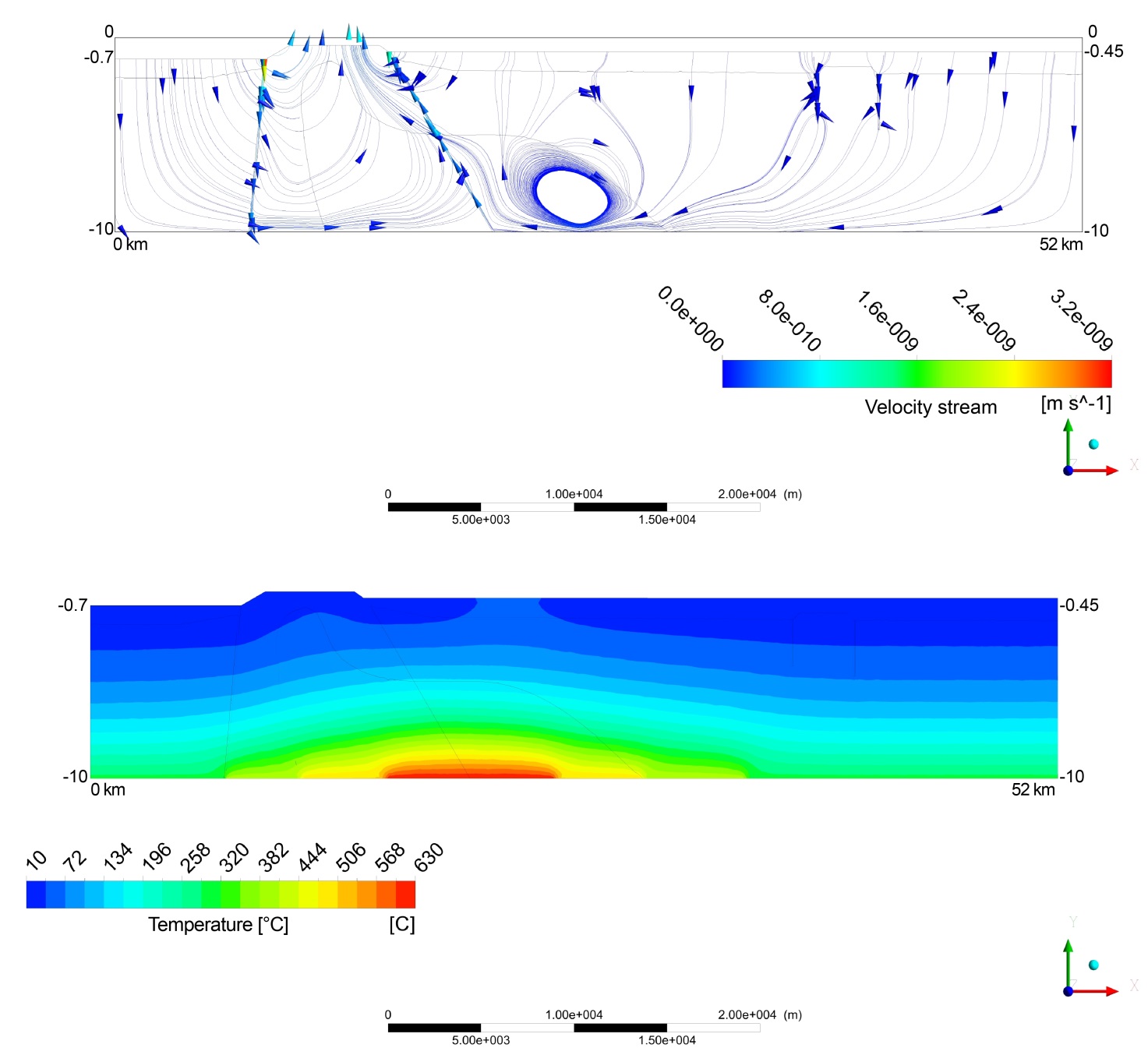


**Supplementary Figure 7 | Testing converge of numerical flow model solution.** Model snapshot at 300 ka. Fluid pathways and flow velocities (top), and temperature distribution (bottom). Model geometry and physical parameter as in Fig. 6.


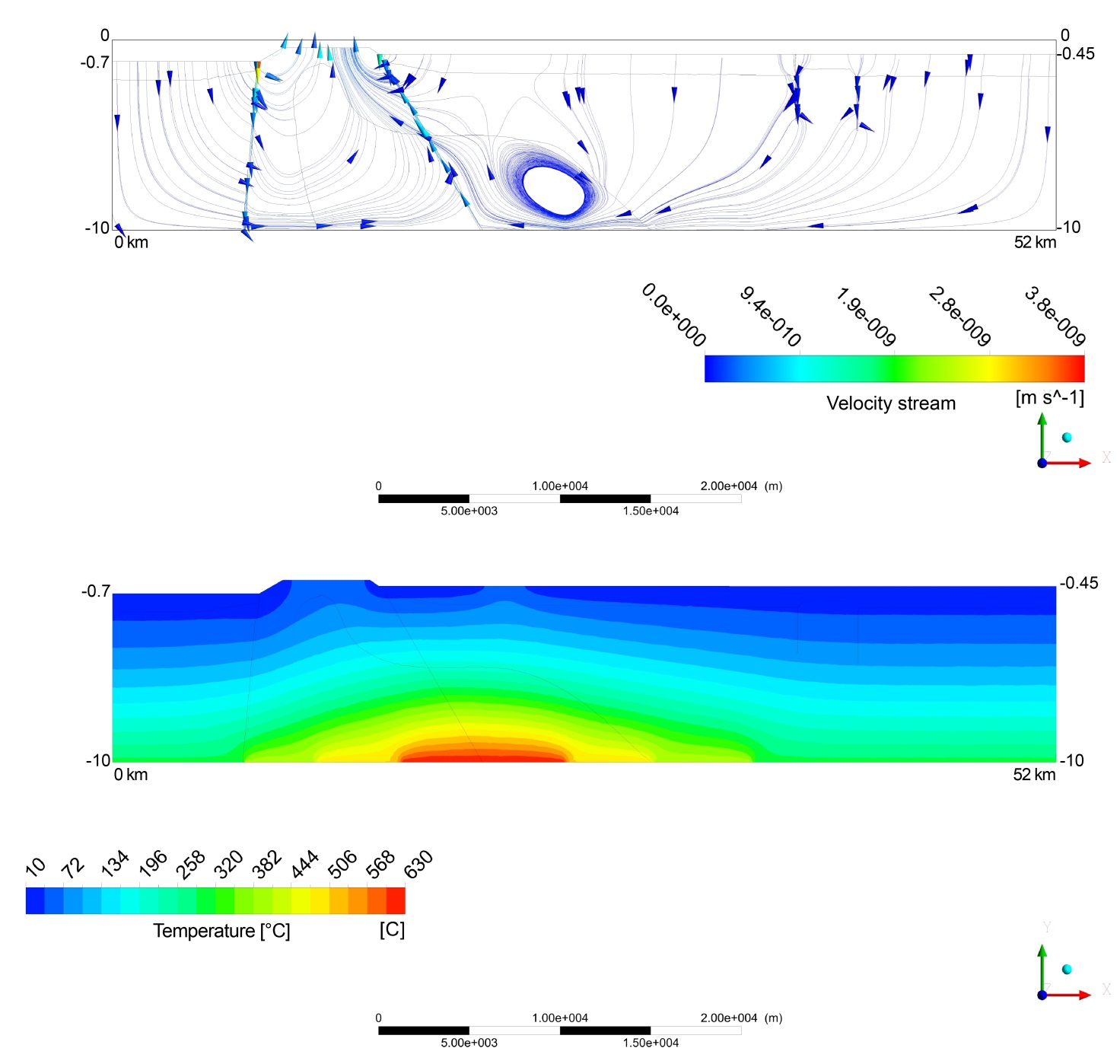


**Supplementary Figure 8 | Testing converge of numerical flow model solution.** Model snapshot at 0.5 Ma. Fluid pathways and flow velocities (top), and temperature distribution (bottom). Model geometry and physical parameter as in Fig. 6.


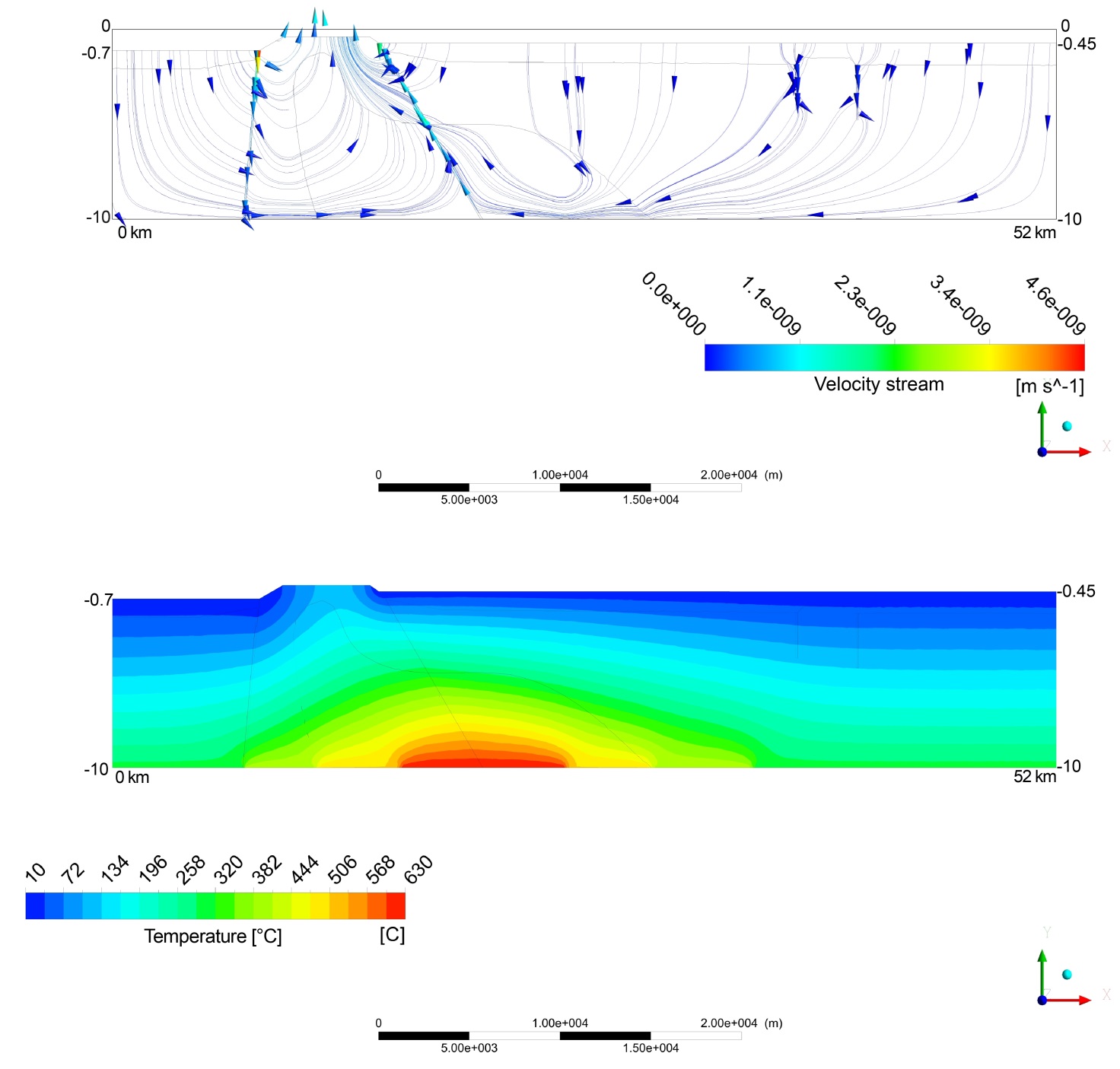


**Supplementary Figure 9 | Testing converge of numerical flow model solution.** Model snapshot at 1.0 Ma. Fluid pathways and flow velocities (top), and temperature distribution (bottom). Model geometry and physical parameter as in Fig. 6.

**References**

1. Vogel, H. Das temperature-abhängigketsgesetz der viskosität von flüssigkeiten. *Phys. Z.* **22**, 645-646 (1921).
2. Ozbek, H., ve Phillips, S. L. Thermal conductivity of aqueous sodium chloride solutions from 20 to 330 °C. *Journal of Chemical and Engineering Data* **25**, 263–267 (1980).
